# Localizing Clinical Patterns of Blast Traumatic Brain Injury Through Computational Modeling and Simulation

**DOI:** 10.3389/fneur.2021.547655

**Published:** 2021-05-20

**Authors:** Scott T. Miller, Candice F. Cooper, Paul Elsbernd, Joseph Kerwin, Ricardo Mejia-Alvarez, Adam M. Willis

**Affiliations:** ^1^Computational Solid Mechanics & Structural Dynamics, Sandia National Laboratories, Albuquerque, NM, United States; ^2^Terminal Ballistics Technology, Sandia National Laboratories, Albuquerque, NM, United States; ^3^Department of Neurology, Brooke Army Medical Center, Fort Sam Houston, TX, United States; ^4^Department of Mechanical Engineering, Michigan State University, East Lansing, MI, United States

**Keywords:** traumatic brain injury, blast, cavitation, simulation, computation, interfacial injury

## Abstract

Blast traumatic brain injury is ubiquitous in modern military conflict with significant morbidity and mortality. Yet the mechanism by which blast overpressure waves cause specific intracranial injury in humans remains unclear. Reviewing of both the clinical experience of neurointensivists and neurosurgeons who treated service members exposed to blast have revealed a pattern of injury to cerebral blood vessels, manifested as subarachnoid hemorrhage, pseudoaneurysm, and early diffuse cerebral edema. Additionally, a seminal neuropathologic case series of victims of blast traumatic brain injury (TBI) showed unique astroglial scarring patterns at the following tissue interfaces: subpial glial plate, perivascular, periventricular, and cerebral gray-white interface. The uniting feature of both the clinical and neuropathologic findings in blast TBI is the co-location of injury to material interfaces, be it solid-fluid or solid-solid interface. This motivates the hypothesis that blast TBI is an injury at the intracranial mechanical interfaces. In order to investigate the intracranial interface dynamics, we performed a novel set of computational simulations using a model human head simplified but containing models of gyri, sulci, cerebrospinal fluid (CSF), ventricles, and vasculature with high spatial resolution of the mechanical interfaces. Simulations were performed within a hybrid Eulerian—Lagrangian simulation suite (CTH coupled *via* Zapotec to Sierra Mechanics). Because of the large computational meshes, simulations required high performance computing resources. Twenty simulations were performed across multiple exposure scenarios—overpressures of 150, 250, and 500 kPa with 1 ms overpressure durations—for multiple blast exposures (front blast, side blast, and wall blast) across large variations in material model parameters (brain shear properties, skull elastic moduli). All simulations predict fluid cavitation within CSF (where intracerebral vasculature reside) with cavitation occurring deep and diffusely into cerebral sulci. These cavitation events are adjacent to high interface strain rates at the subpial glial plate. Larger overpressure simulations (250 and 500kPa) demonstrated intraventricular cavitation—also associated with adjacent high periventricular strain rates. Additionally, models of embedded intraparenchymal vascular structures—with diameters as small as 0.6 mm—predicted intravascular cavitation with adjacent high perivascular strain rates. The co-location of local maxima of strain rates near several of the regions that appear to be preferentially damaged in blast TBI (vascular structures, subpial glial plate, perivascular regions, and periventricular regions) suggest that intracranial interface dynamics may be important in understanding how blast overpressures leads to intracranial injury.

## Introduction

Traumatic brain injury is a “signature wound” of modern conflict with estimates of over 320,000 service members wounded with traumatic brain injuries (TBI) (1) during Operation Iraqi Freedom and Operation Enduring Freedom. Head trauma accounts also for significant morbidity and mortality in combat, accounting for 38% of immediate fatalities and 53% of those who die prior to admission to a medical treatment facility (2). Fifty-two percent to 65% of service members hospitalized for head trauma were exposed to blasts (1, 3). It is clear that blast TBI poses a significant risk to service members and larger scientific efforts have started to unravel the complex pathophysiology of TBI. However, because of the difficulties in carefully controlling mechanical deformations from blast loading in laboratory settings, the underlying linkage of tissue deformation to tissue injury—remains largely unknown.

The physical mechanism by which blast pressure waves can cause intracranial damage is complex and likely is the interplay between multiple physical mechanisms. In order to differentiate blast injury components, there exist four natural divisions: primary injury—blast pressure wave transmitting into skull; secondary injury—penetration of projectiles through the skull and brain; tertiary injury—acceleration / deceleration from blast; and quaternary injury—thermal, chemical, other injuries to head, face, scalp, and respiratory tract. Primary blast injury is hypothesized to be unique (relative to other mechanisms of TBI), this is possibly secondary to the high frequency stress waves interacting with the human head not experienced in traditional blunt impact TBI. In order for primary pressure waves to cause injury, extracranial pressure must be transmitted into the human brain. One possible transmission mechanism is *via* skull orifices—however experimental and computational evidence do not support this being a dominant mechanism (4). Another possible mechanism is direct transmission of pressure waves through skin/skull/CSF into the brain, which is well-supported by experimental and computational evidence (4). In conjunction with direct transmission of pressure waves into human skull, it is hypothesized that blast waves deform the human skull, which is stored as elastic strain energy, and then transmitted into CSF *via* skull oscillations leading to additional intracranial stress waves (both pressure and shear) (5). Reviews of existing literature support that skull oscillations is also a plausible mechanism in blast traumatic brain injury (4). Because of the interaction of the blast pressure wave with the skull, it is also hypothesized that the skull acts as a high frequency filter (6, 7) for incoming stress waves and removes the highest frequency components from being transmitted into the brain.

Once intracranial, pressure and shear waves are transmitted through various tissue interfaces where it is hypothesized that distinct changes in density could lead to spallation as well as cavitation bubble formation (for example in the CSF spaces) (8, 9). The inception, growth, and collapse of these bubbles would result in disruption to cerebral and vascular tissue. Direct *in vivo* evidence of bubble formation during blast exposure is lacking. However, both computational and experimental cadaveric or tissue surrogate models (8, 10–12) do support the hypothesis that cavitation could occur intracranially during blast exposure. An additional hypothesis has been proposed that thoracic transmission intracranially of high pressure waves *via* extra-cranial vasculature and CSF spaces may also lead to intracranial injury. However, support for such a hypothesis is essentially mixed from existing experimental *in vivo* animal models (4, 13). Nevertheless, once pressure and stress waves are intracranial, it is relatively unclear how specific components of these stresses and strains lead to nervous tissue injury.

In order to better understand how blast waves could produce traumatic injury, it is most helpful to review the known clinical and neuropathologic evidence from humans exposed to blast waves. Acutely, there are several features of blast TBI that appear to differentiate it from conventional blunt TBI. Ling et al. highlighted key differences in blast TBI (relative to blunt) based upon clinical experience of military neurosurgeons and neurointensivists who treated acute, severe blast TBI cases in the deployed military environment (14). Firstly, blast TBI has clinically significant cerebral edema (minutes to hours) after exposure. Secondly, blast TBI has more significant subarachnoid hemorrhage and pseudo-aneurysms—which implies disruption of the subarachnoid intracranial vessels. Thirdly, there were reports of delayed arterial vasospasm in settings where subarachnoid blood was not prominent—again an atypical finding.

This overall pattern of clinical injury is also consistent with more recent clinical literature from Thailand (15). Furthermore, multiple case series of wounded service members also support the hypothesis that there is a predilection for intracranial vascular injury following blast (16–18). A possible unifying feature of these injury patterns is vascular disruption at both the microscale—leading to cerebral edema—as well as at the macroscale—leading to subarachnoid hemorrhage, pseudoaneurysm, and delayed vasospasm.

Neuropathologic studies have helped further define the injury pattern of blast traumatic brain injury. In a carefully constructed case series which compared service members with blast TBI to control TBI cases, there appears to be unique astroglial scarring patterns in blast (19). In this study, diffuse gliosis was found at the following mechanical interfaces: sub-pial glial plate—cerebrospinal fluid (CSF) interface, perivascular regions, periventricular regions, as well as at the gray-white interface [where there is an ~20% shear moduli mismatch (20)].

A possible unifying interpretation of the unique features of blast TBI is that it is an injury of material interfaces. However, testing such a hypothesis is challenging. Experimental animal studies of blast TBI have yielded inconsistent findings across the various sizes and skull anatomies studied (21–24) and have not demonstrated interfacial scarring or the significant vascular injury and diffuse cerebral edema found in humans. Experiments of gelatin phantoms encased in various models of skulls have provided insight into intracranial dynamics during blast traumatic brain injury (10–12), however these models do not include models of the key anatomy which appears to be injured in blast exposure –vascular structures, ventricles, sulci/gyri, and gray-white interfaces. Over the past two decades as computational resources have improved, there has been a trend of iterative improvement of the details of human anatomy simulated to investigate blast TBI (5, 8, 20, 25–27). However to date, there has not been a dedicated effort to increase spatial resolution of computational simulations to explicitly model the regions where it appears blast TBI is most injurious, the intracranial mechanical interfaces. Given our hypothesis that blast TBI injury is an interface injury, we take a novel simulation approach –simulating an idealized model of human anatomy with high spatial resolution to ensure an accurate definition of interface mechanics between relevant intracranial structures.

## Materials and Methods

### Surrogate Head Model

**Figure 1:**
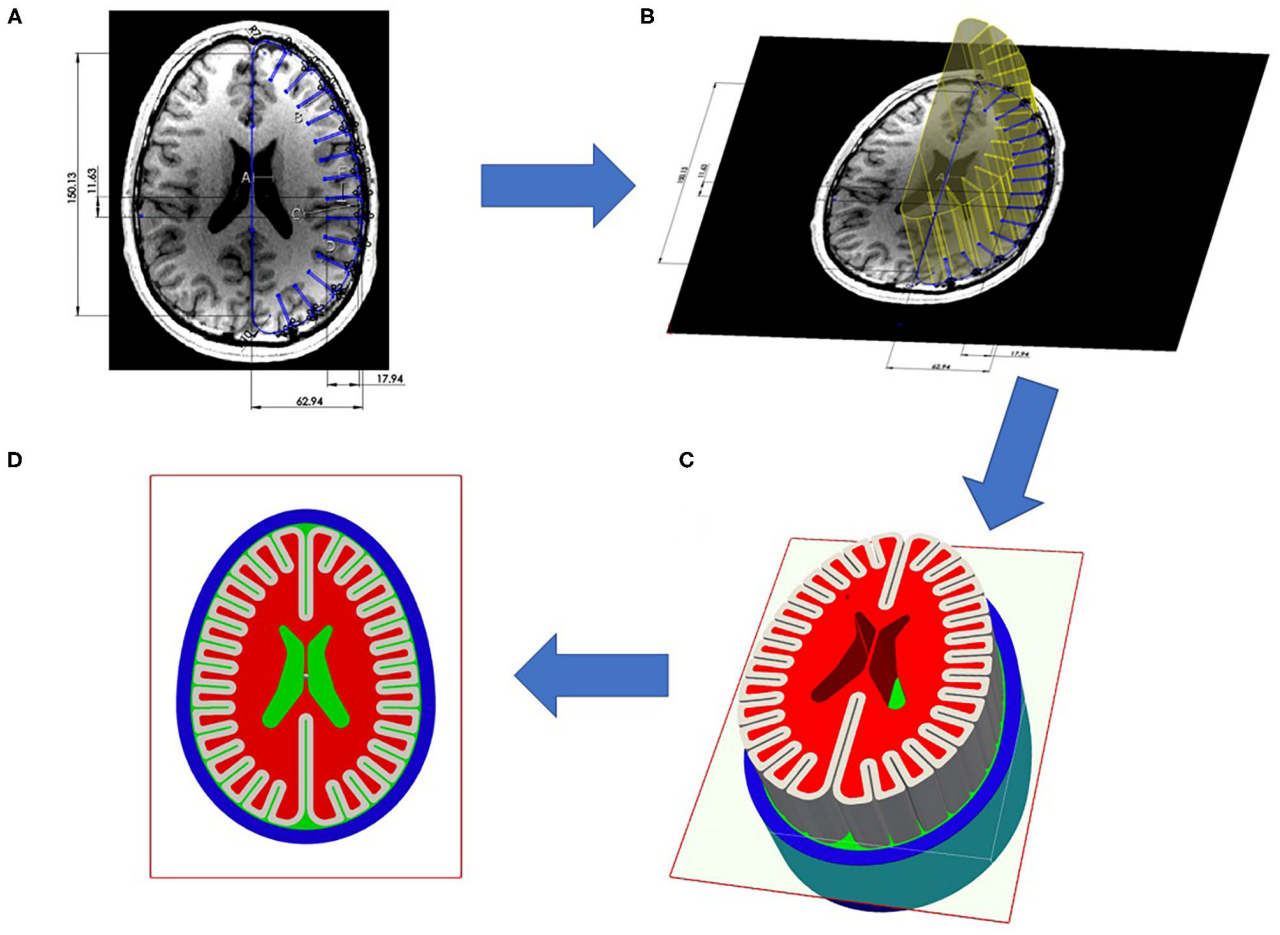
Construction of test object from principle investigators brain magnetic resonance imaging (MRI): **(A)** Axial section of principal investigators brain with measurements of length scales of width, length, gyral thickness, gray matter thickness, sulci width, sulci depth, average skull thickness. (Not shown is coronal section to determine height length scale). Using obtained length scales from axial section, CAD developed canonical axial brain slice. **(B)** This axial brain slice was visually compared to original axial slice to ensure gross representation of dominant length scales, then slice was extruded to match the height of human brain. **(C)** This extruded axial prism was meshed to create three dimensional test object. **(D)** Results presented are two dimensional projections of the element values from the mid-plane of test object.

A three-dimensional (3D) geometric representation was derived from an idealized human brain axial section as depicted in Figure 1. This axial section was constructed *via* computer aided drafting (CAD) software (SolidWorks, 2018) and then extruded to match the overall height of a human brain. The values of height, width, thickness as well as the ratios and dimensions of sulcal depth to gyral width, sulcal width, CSF layer thickness, gray / white matter, ventricle size were compared to principal investigator's (35-year-old-male, healthy) brain magnetic resonance image (MRI) imaging. The aim of this geometry was to model the key phenomenology of intracranial interface dynamics, yet was simplified enough to provide mechanistic insight into blast traumatic brain injury. The derived anatomy is a generalized representation / notional model of a human brain to study phenomenology and does not reflect any personalized attributes. Although geometrically simple, the uniqueness of this model is the high spatial resolution in which we meshed this simplified anatomy, in order to ensure that simulations resolve the appropriate interface mechanics. Additionally, this design can be fabricated into experimental phantoms for future efforts to support the results of these computational simulations. The geometry was reviewed and verified by a board-certified neurologist who confirmed the relative size and shape of the human head was maintained in the brain phantoms while maintaining the dominant length scales of the gyri/sulci. See Figure 1.

### Computational Model and Materials

#### Meshed Phantom

Using the developed CAD model, Cubit (28) software was used to create all hexahedral finite element meshes of the surrogate head models. The mesh design was optimized to resolve the thin gray matter layer and CSF layer, each employing a minimum of 5 elements through the thickness. Without vasculature, the model consists of 2.3 million elements, with a more detailed breakdown of mesh statistics given in Table 1. The vascular model also included small diameter channels (0.6, 0.8, 1.2, and 1.6 mm) which were surrounded by a layer of five elements to model a vascular wall 10% of the entire vessel diameter. This highly refined vasculature model contains 11.8 million elements and details are provided in Table 2. The element “size” provided is a measure of the element diameter and is directly related to the fidelity of the computational results. Typical linear finite element methods (as used here) converge quadratically as the element size is decreased (29).

**Table 1:** Mesh statistics without vasculature.

	**Number of elements**	**Minimum element size (cm)**	**Maximum element size (cm)**	**Average element size (cm)**
Skull	820,432	5.051e-01	9.999e-01	9.475e-01
White	531,824	6.368e-01	9.995e-01	9.399e-01
Gray	665,296	5.956e-01	9.977e-01	9.078e-01
CSF	351,152	5.051e-01	9.995e-01	8.869e-01
Entire model	2,368,704	5.051e-01	9.999e-01	9.257e-01

**Table 2:** Mesh statistics with vasculature.

	**Number of elements**	**Minimum element size (cm)**	**Maximum element size (cm)**	**Average element size (cm)**
Skull	1,889,374	7.717e-03	1.000e+00	3.780e-01
White	6,466,560	1.014e-02	9.272e-01	2.752e-01
Gray	2,325,060	1.742e-01	7.449e-01	4.582e-01
CSF	781,190	1.443e-02	9.999e-01	5.123e-01
Vessel interiors	426,240	8.936e-03	1.276e-01	3.325e-02
Entire model	11,888,424	7.717e-03	1.000e+00	3.342e-01

#### Air Blast Domain

Blast loading onto the head model is accomplished with the shock wave physics code CTH (30) CTH is a multidimensional finite volume code capable of accurately resolving shock wave propagation. Air is modeled *via* a SESAME tabulated equation of state in CTH. A uniform grid of 0.25 mm cubic cells is used to resolve the shock wave propagation in air. Shock loading is generated from an energized slab of air tuned to give a specific overpressure and Friedlander shaped shock profile with overpressure durations of 1 ms, see Table 3. Described in Table 3 are the thermodynamically consistent CTH conditions and the required stand-off distances to generate the Friedlander profiles of the specified duration (1 ms) and overpressures (150, 250, and 500kPa). The blast characteristics were not aimed to directly model any particular blast exposure. The choice of the 1 ms overpressure duration is convenient but representative of smaller blasts at close range—pipebombs or rocket propelled grenade blasts. This shorter duration also improved computational efficiency secondary to shorter simulations and smaller computational domains, furthermore it was chosen to match the overpressure durations in upcoming experimental validation studies. Amplitudes where chosen such that reflected waves overpressures would approximately achieve the levels in which NHP studies began demonstrating changed in cerebrospinal fluid injury biomarkers (31). A reflective boundary condition must be used adjacent to this energized air to direct the energy toward the test object. All other boundaries utilize an outflow boundary condition. Simulations were run to determine a sufficiently large domain size to ensure that boundary conditions do not affect the mechanical response of the head in the time periods considered herein. Three blast scenarios were simulated (see Figure 3), front blast, side blast, and wall blast. In order to investigate if a more complex blast wave (which included reflections / mach stem) altered the phenomenology of blast TBI, we included a wall at an arbitrary distance / angle to the incident blast. The wall blast was identical to the front blast, except for the inclusion of a reflecting surface 6.3 cm from side of head at angle of 26.5° off from direction of blast.

**Table 3:** Initial conditions for front blast.

**Overpressure**	**Density (g/cm^**3**^)**	**Energy (ergs/g)**	**Pressure (dyne/cm^**2**^)**	**Energized air thickness (cm)**	**Standoff distance (cm)**	**Domain size (cm^**3**^) Front Blast /Side Blast**
150 kPa	3.5815e-3	0.70547e10	5.3912e6	4.7	45	60x85x50 / 82.5x58.75x50
250 kPa	4.6213e-3	0.85227e10	9.5101e6	3.6	35	60x75x50 / 72.75.x58.75x50
500 kPa	5.8544e-3	1.22377e10	20.0276e6	3.4	30	60x70x50 / 67.25.x58.75x50

#### Computational Head Model

**Figure 2:**
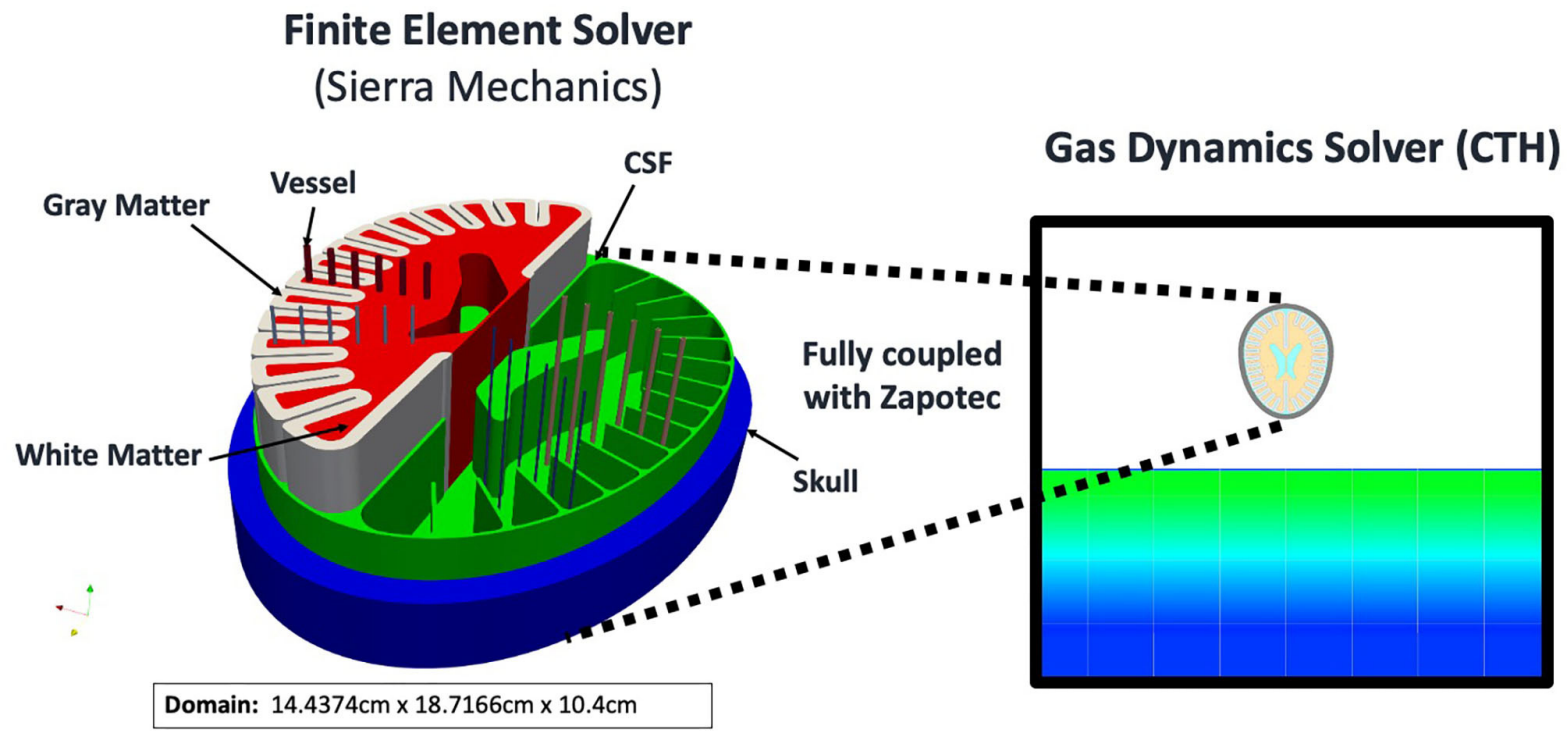
Surrogate head model and embedding finite element solver into gas dynamics solver.

Finite element simulations of the surrogate head model (and all intracranial contents) were conducted using the Sierra Solid Mechanics (32) software from Sandia National Laboratories. We use this Lagrangian finite element software to explicitly integrate the dynamical equations of motion for a 3-dimensional solid. Material models were used for the constituent parts of skull, gray matter, white matter, and CSF. For the skull, a hyperelastic solid (see Table 4) was used. There is significant variation in skull properties reported and our parameters fit within experimentally derived values (33). Our gray matter and white matter models were modeled *via* a Tillotson-Brundage (27, 34) equation of state (EOS) for dilatational response (Table 5) while using viscoelastic model for deviatoric response (Table 6) (27). Reported values of the parameters to model white and gray matter also vary significantly (35) between study, species, and age of specimen, thus we used both the meticulously derived values of Taylor (27) and simulated with values reduced by an order of magnitude to ensure stability of results to material parameters. CSF and blood are indistinguishable in our simulations. They are both modeled using the same Tillotson-Brundage equation of state (EOS) for dilatational response (Table 5) which had no deviatoric strength. We did not include separate tissue models for vessel walls, thus our model of vascular structures contained only a fluid filled cavity embedded within the brain model, see Figures 2 and **5**. Thus, our vascular models were fluid channels embedded in the brain without any surrounding endothelium. Although this model will not resolve the important mechanical interactions of the mechanics within vascular tissue (which could provide mechanical insight into the development of pseudoaneurysms or vasospasm), it will capture the fluid-structure interaction and any possible cavitation events within the vasculature.

**Table 4:** Elastic material properties of skull.

**Material**	**Density (g/cm^**3**^)**	**Young's Modulus (MPa)**	**Poisson's ratio**
Skull	1.21	2000	0.40

**Table 5:** Tillotson-Brundage Equation of State (EOS) parameters for intracranial material.

**Material**	**Density (g/cm^**3**^)**	**T-B EOS parameter A (kPa)**	**T-B EOS parameter B (kPa)**	**Cavitation threshold (kPa)**
White matter	1.04	2.18e6	13.25e6	−300
Gray matter	1.04	2.18e6	13.25e6	−300
CSF	1.04	2.18e6	13.25e6	−200

**Table 6:** Viscoelastic properties of white and gray tissue.

**Material**	**Short-term shear modulus G_**0**_ (kPa)**	**Long-term shear modulus G_**∞**_ (kPa)**	**Decay constant β (s^**−1**^)**
White matter	33.2	7.8	40
Gray matter	27.6	6.4	40

**Figure 3:**
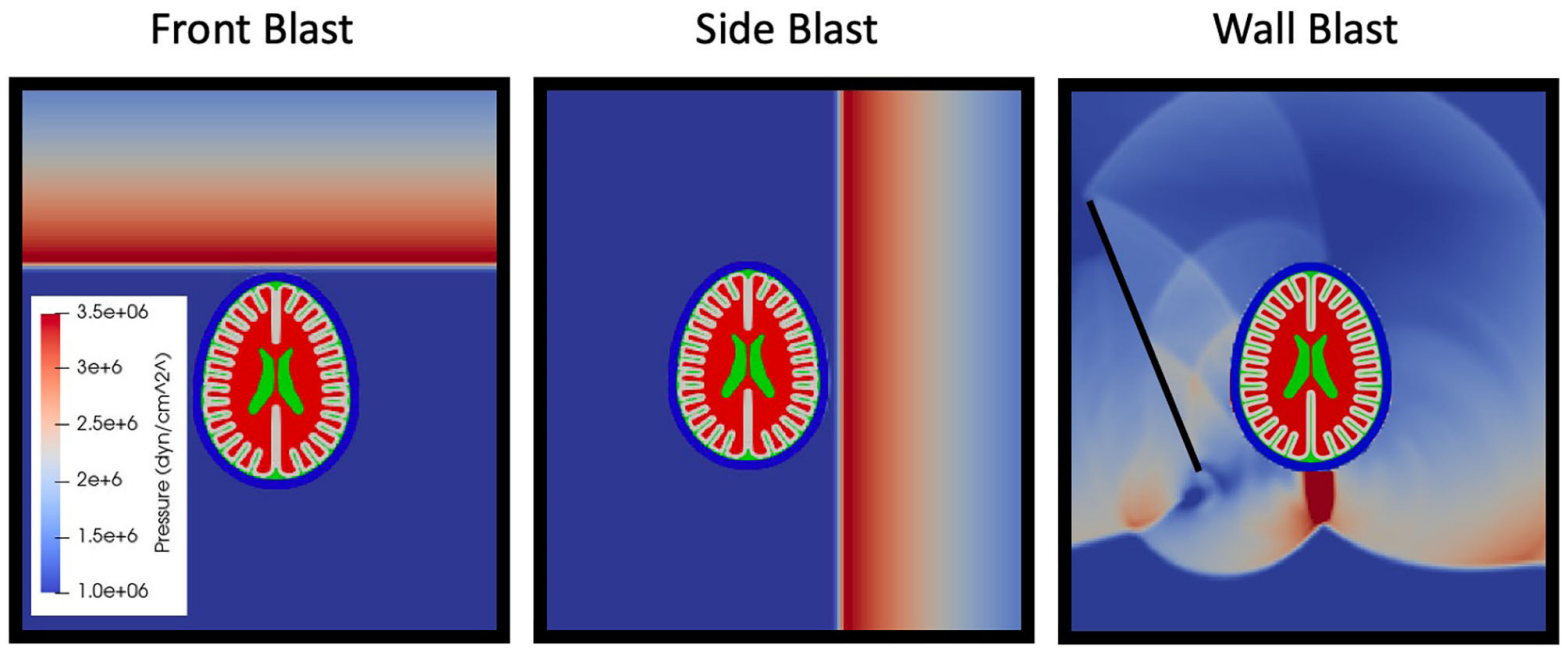
Simulation scenarios of front, side, and wall blast. Later time simulation of wall shown to highlight complexity of reflected waves between skull and head.

#### Air Blast—Head Model Coupling

Blast-on-structure simulations are challenging due to the need to couple two different physical domains, air with a spatial description and the head with a material description. Adding to this complexity is the technical challenge of coupling the different software pieces. The Sierra Zapotec (36) tool couples the air blast in CTH to the finite element simulation in Sierra Solid Mechanics yielding a powerful yet scalable computational framework capable of simulating highly resolved computational meshes Figure 2.

#### Cavitation Modeling

Cavitation events are modeled using a minimum pressure approach, similar to other TBI studies (5, 8, 12). Thus, once a defined minimum pressure / tension is achieved, local material stress will be fixed at that prescribed level—essentially providing a yield point of the material—where further deformation will not further increase material tension. Cavitation rebound occurs when an element's pressure increases above the cavitation threshold, at which point the element is no longer cavitating. No damage modeling mechanisms are being modeled here, and material response after cavitation rebound is unchanged by the cavitation event itself. That is, an element's cavitation history does not impact its instantaneous response.

Cavitation inception at the scale of element size is captured, but the fine details of cavitation bubble growth and subsequent collapse are not resolved. Therefore, cavitated elements can be viewed as markers of where cavitation may occur, but would require more localized studies of cavitation events to understand the effects on surrounding tissue. Thresholds were set at gauge pressures of −200 kPa for CSF (5, 8, 12) and −300 kPa (37) for intracranial tissue.

## Results

We performed 20 simulations. Two sets of nine simulations without vasculature were performed with overpressures of 150, 250, and 500 kPa with three configurations of front blast, wall blast, and side blast. Material properties of initial simulates were those derived by Taylor et al. (20). For all simulations, peak pressures and strains all occurred during the first 4 ms of simulation—thus all non-vascular simulations were performed to 6 ms. The second set of nine simulations was performed with shear properties reduced by an order of magnitude. Two additional simulations were performed, one with the front 150 kPa blast upon the highly resolved vascular mesh, and another with an increased skull stiffness. Simulations that did not include vasculature were performed on the Sandia High Performance Computing cluster Skybridge. Using 256 computational cores, 6 ms of simulation time took ~6 h of wall time. The vascular model simulation was also performed on Skybridge using 1,024 cores with 2.5 ms of simulation time costing ~30 h of wall time.

### Canonical Simulation Results

**Figure 4:**
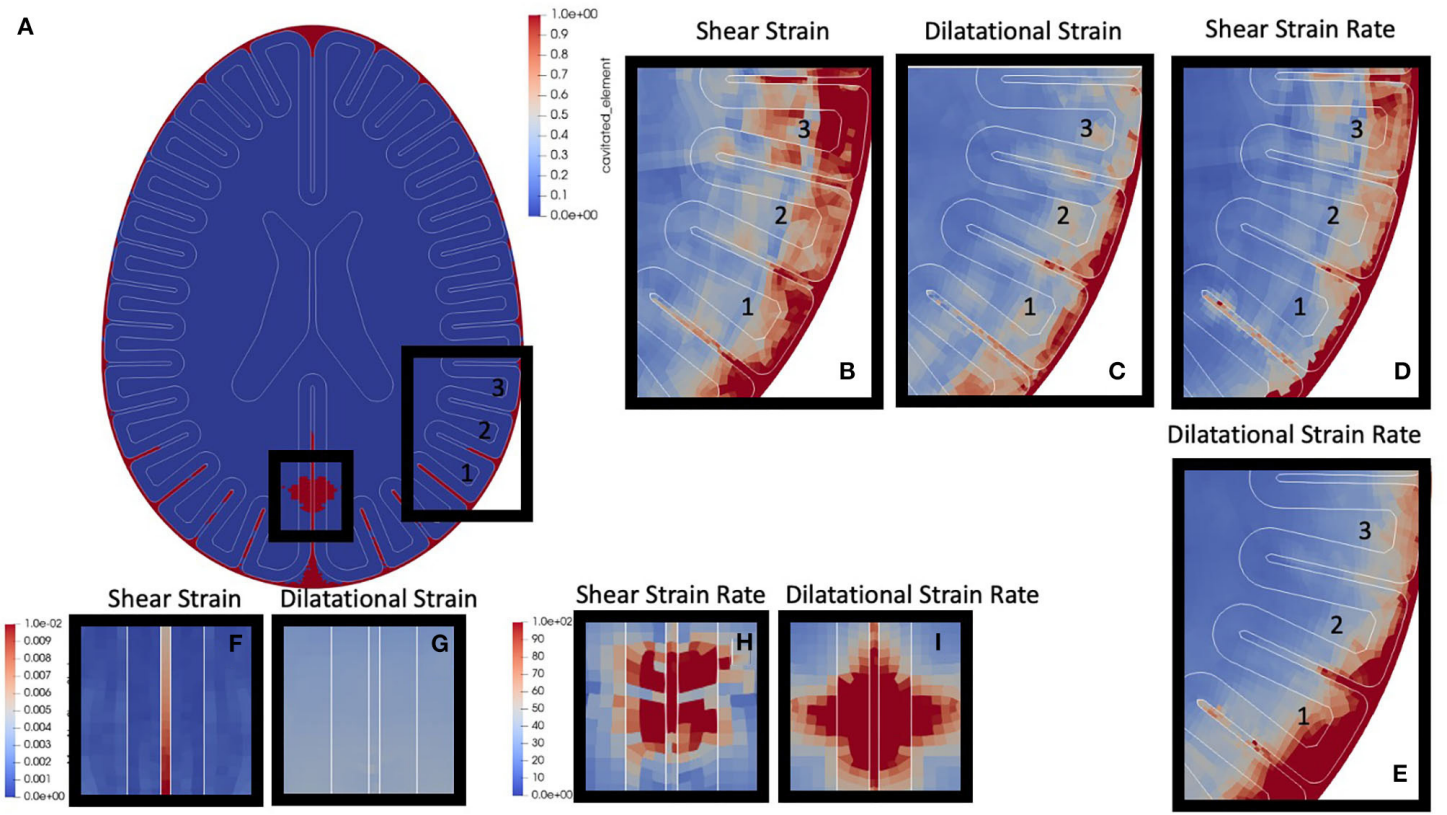
Canonical Blast Results: **(a)** Cavitation events are highlighted in red. This simulation highlights both parenchymal cavitation events (smaller box) and sulcal cavitation events (larger box). **(b)** Zoomed shear strain around three gyri of interest, label “1,” “2,” and “3.” **(c)** Dilatational strain. **(d)** Shear strain rate. **(e)** Dilatational strain rate. **(f)** Shear strain region of parenchymal cavitation. **(g)** dilatational strain in region of parenchymal cavitation. **(h)** Shear strain rate in region of parenchymal cavitation **(i)** dilatational strain rate in region of parenchymal cavitation.

A 250 kPa front blast was performed (see Figure 4, skull has been removed to aid visualization of interface mechanics). Output of the simulation included any cavitation event throughout the course of the simulation, and element-wise maximum values of: absolute value shear strain, dilatational strain, absolute value shear strain rate, and dilatational strain rate. Maximum and minimum intracranial pressures were also output but are not shown. Simulations have three key regions of interest: the skull-CSF-brain interface, the peri-gyral regions, as well as brain parenchymal cavitation zones.

The first region of interest is the skull-CSF-brain interface. The surrounding CSF layer adjacent to the skull shows cavitation (red regions in Figure 4A) almost completely enveloping the outer portions of the brain. Within the CSF layer, there is also local maximization of absolute shear strain, dilatational strain, and the respective strain rates. These high strains and strain rates also propagate radially inward into the gray / white matter closest to the skull (shown in Figures 4B–E).

The next region of interest is the peri-gyral regions. Highlighted in Figure 4A with the larger black box, it is clear there are cavitation events down into the depths of the sulci (lower portion of gyrus “1,” and partway down the lower sulci of gyri “2,” while very little into lower portion of gyrus “3”). Gyral strain rates adjacent to cavitation events (shear > dilatation) – seen in the lower sulci of gyrus 1 (Figures 4D,E) – have local maxima, while strains do not show this same pattern (Figures 4B,C). Interestingly, gyral strains (Figure 4B, gyrus 3) appear maximum near the gray-white interface in gyri which do not cavitate.

The third region of interest are the parenchymal cavitation zones, such as those highlighted in the small box in Figure 4A. Tissue strains (Figures 4F,G) do not show local maxima, however these cavitation events do co-localize with high, focal, strain rates (Figures 4H,I) within the parenchymal tissue.

### Vascular Cavitation Events

**Figure 5:**
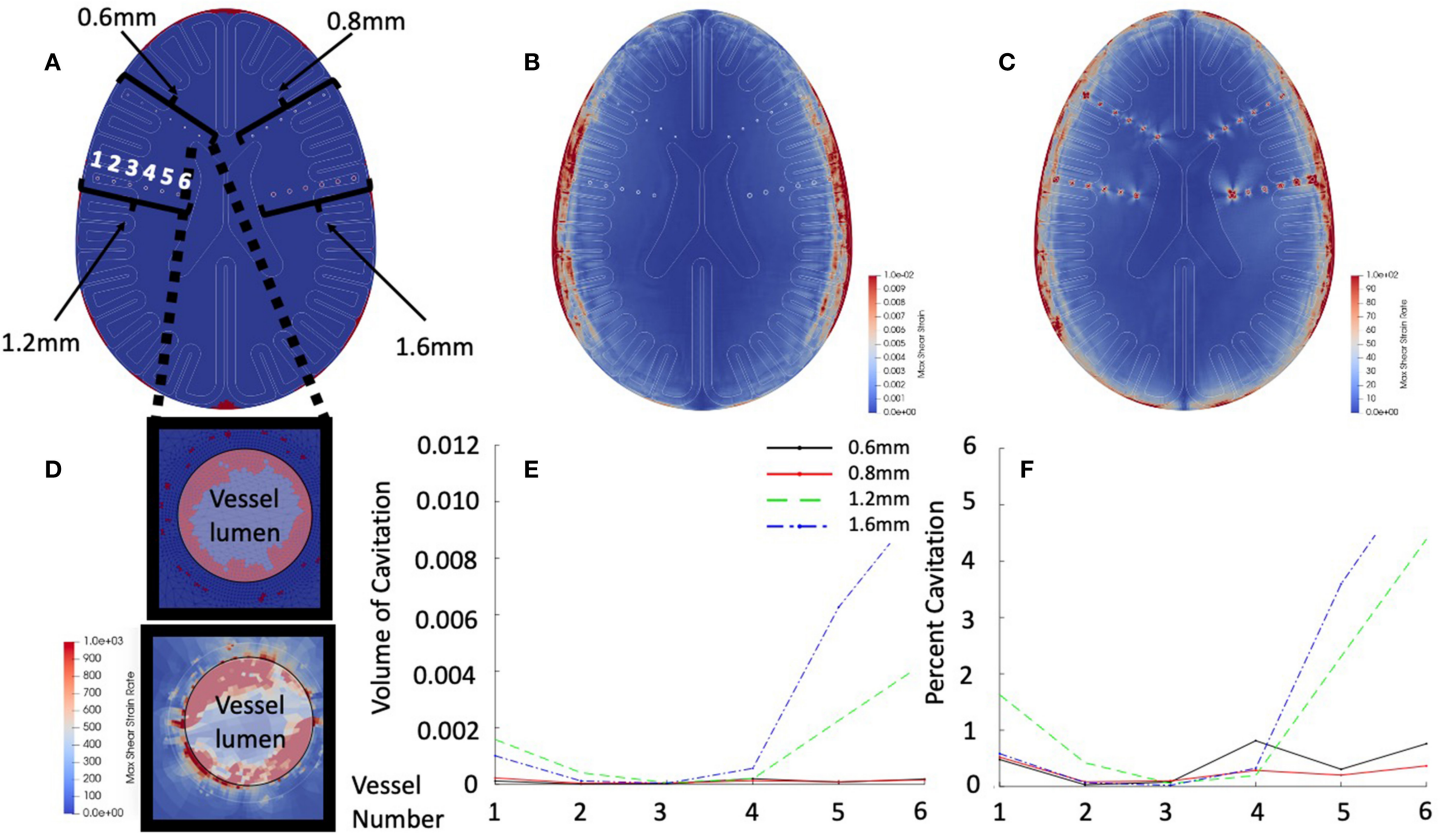
Vascular simulation results at 2.5 ms. **(A)** Cross-section of vascular model. Highlighted are the vessel models of diameters of 0.6, 0.8, 1.2, and 1.6 mm. Vessels are numbered from lateral to medial position. **(B)** Shear strain maximum in early time simulation. **(C)** Shear strain rate in early time simulation. **(D)** Intravascular cavitation events of 0.6 mm vessel –position 6 (top). Focal strain rates within vessel wall (bottom). **(E)** Total vessel cavitation volume as function of position and diameter. **(F)** Percent of vessel volume cavitation as a function of position and diameter.

In order to investigate the dynamics of small vascular structures within the human brain, a high-resolution finite element mesh (see Figure 5A for geometry, and Figure 5D for mesh resolution), was simulated. This model was sufficiently resolved to capture the structural mechanics of these vascular structures. Simulation results were focused on early time (<2.5 ms) dynamics. Figure 5A shows a cross-section of the skull model with embedded vascular elements. Four different vascular sizes were embedded (diameters 0.6, 0.8, 1.2, and 1.6 mm) with varying distances from the periphery of the skull (labeled “1” – “6,” with vessel “1” of each set being closest to the periphery of the brain). Figure 5B shows that the inclusion of the vascular structures does not dramatically alter intraparenchymal strain patterns, however Figure 5C shows the dramatic strain rates surrounding these vascular structures. Figure 5D (top) shows the prediction of cavitation events predominantly within the vascular structures, while Figure 5D (bottom) shows the focal absolute value shear strain rates both within the vessel (highlighted with white) and in the adjacent brain tissue. Notice that strain rates are in excess of 10^3^. Figure 5E plots the volume of cavitated elements contained within each vessel as a function of position and size while Figure 5F normalizes the amount of cavitated elements to the total vascular volume. Luminal cavitation occurred within all of the vascular structures, however, both the total volume of cavitation and the relative volume of cavitation appear to be functions of position (cavitation most prominent closer to CSF/tissue interface, especially near the ventricles) and vessel size (largest-vessel cavitation was more prominent in volume and relative percentage of volume than in vessels with small diameter).

### Patterns of Cavitation

**Figure 6:**
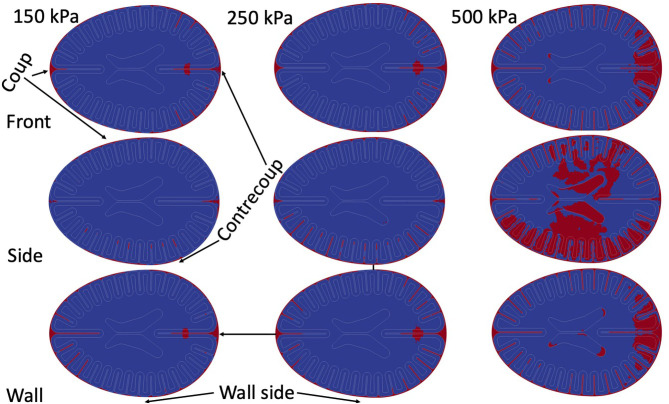
Cumulative cavitation events as a function of blast exposure over entire 6 ms simulation.

Multiple simulations were performed to test for the generalizability of results to multiple blast scenarios. Figure 6 demonstrates the results of the simulations. Because of the co-location of cavitation events and patterns of strain rates, only cavitation results are presented as a function of blast parameters. For all of the simulations, the pattern of strains remained essentially unchanged in the three regions of interest and were grossly consistent with overpressure 250 kPa front blast. For the strains / strain rates nearest the skull, the depth of significant strains mildly increased with larger overpressure exposures.

When focusing on the peri-gyral regions, for the 150 kPa blasts, all simulations demonstrated deep sulcal cavitation with the front and side blast cavitation events localizing in the contrecoup regions while the wall blast demonstrated more frontal cavitation events (wall side more prone to exhibit cavitation than opposite of wall). The 250 kPa blasts all demonstrate the same key features of the 150 kPa blasts but with more prominent cavitation events.

Small regions of parenchymal cavitation occur in all of the front blast / wall blasts around what would be analogous to the intrahemispheric fissure—nearest the contrecoup location. However, at 500 kPa, exposures demonstrate a new pattern –intraparenchymal cavitation within sulci. Additionally, at 500 kPa intraventricular cavitation becomes prominent near the horns of the ventricles in all of the scenarios.

### Parameter Study

**Figure 7:**
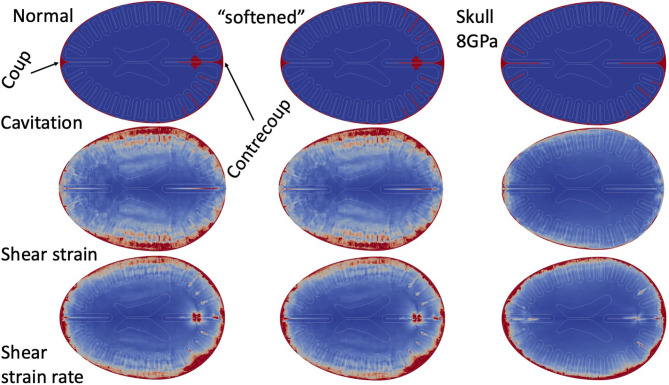
Parameter study. Simulation results (cavitation, shear strain, and shear strain rate) in Normal simulations, shear “softened” simulations, and 8.0 GPa skull.

In order to ensure our results are not sensitive to the particular set of material parameters chosen, we carried out a series of simulation tests: first, we simulated a complete set of overpressures (150, 250, and 500 kPa) for each exposure scenario (front blast, side blast, and wall blast) with shear moduli of the brain set at 10 times less than the values in Table 6. Second, we performed another simulation of 250 kPa—front blast, with Young's Modulus of 8.0 GPa and Poisson ratio of 0.22. Figure 7 shows the cavitation, shear strain, and shear strain rate for the “Normal” parameters of Table 6, the “softened” simulations with brain matter an order of magnitude softer in shear, as well as the “8 GPa” skull using parameters of Table 4. For all simulation results, “Normal” and “softened” results were essentially identical for the metrics shown as well as all other metrics collected. However, while there were some qualitative differences between the “Skull 8 GPa” simulations, they exhibited grossly similar phenomenology. Cavitation was captured within the sulcal depths and remained correlated to the presence of adjacent high cortical strain rates. The more rigid skull showed diffusely high cortical strain rates nearest the skull vs. the patchy distribution found in the other simulations. Cortical strains were diffusely much less for the more rigid skull.

## Discussion

Our key hypothesis—which was supported by clinical and neuropathologic evidence—is that blast traumatic brain injury is primarily an injury at the mechanical interfaces of the brain. In order to explicitly capture the mechanics of these interfaces we developed a unique hybrid computational suite capable of resolving the mechanical interfaces within an idealized human head.

The first key feature of the simulation results is the close physical association extrema of strain and strain rates at the mechanical interfaces of the brain, which also coincide with the anatomic structures observed in brains injured with blast traumatic brain injury. Specifically, the CSF space—where subarachnoid vessels reside—demonstrates significant strain and strain rates. Although we did not explicitly model these structures with the CSF, it is plausible that the existing vascular structures within these high strain / strain rate regions would be disrupted and lead to subarachnoid hemorrhage, pseudoaneurysms, or delayed arterial vasospasm found in severe blast TBI. Additionally, high tissue strain rates are diffusely found on three of the interfaces shown to have astroglial scarring in blast TBI victims—sub-pial glial plate, peri-ventricular, perivascular (19). These two findings—the association of significant tissue deformation to the anatomic injury—may highlight the importance of the interface mechanics in understanding blast TBI injury.

### Linking Blast TBI Pathology to Computational Models

Many of the clinical outcomes and neuropathologic features of blast TBI co-localize to either CSF spaces or adjacent to CSF spaces. Thus, any structure within these regions (if they do not alter the mechanics) would experience these high strains / strain-rates. However, what is more likely is that the presence of the additional mechanical interfaces of vasculature adjacent to the brain-CSF interface (CSF-vessel interface, vessel tissue interfaces, blood - vessel interface), would also experience further high strains / strain rates and thus would be further prone to injury than implied by our simulations. Subscale simulations, as well as carefully constructed *in vitro / in vivo* experiments will be needed to further investigate the mechanical interaction of vascular structures and resultant pathophysiology under blast loading conditions. Subarachnoid hemorrhage is a prominent component of blast TBI and is highlighted in multiple reviews from clinicians and centers with experience of blast traumatic brain injury (14, 15). Subarachnoid hemorrhage is rupture / injury of the vascular structures suspended within the CSF space.

Pseudoaneurysms, which are injuries to vessels that do not result in vascular rupture are also common following blast (14, 16, 18). The presence of cavitation and high strain-rates within the CSF space could plausibly be a mechanism by which these vascular structures are injured and result in subarachnoid hemorrhage and pseudoaneurysm. Delayed cerebral arterial vasospasm is also prominent in blast—even in the absence of subarachnoid hemorrhage (16). Interestingly, *in vitro* studies of endothelial cells exposed to high strain rates switch to a contractile state—with a similar time delay of contraction to the onset of post-blast arterial vasospasm (38). However, we only explicitly modeled the adjacent tissue to the CSF space, specifically the sub-pial glial plate. Our simulations do localize high strain rates (associated with cavitation) to the sub-pial glial plate. This same interface also shows significant astroglial scarring in neuropathological analysis of blast victims (19) which may share a common mechanism of CSF cavitation and the high surface strain-rates in adjacent tissues.

Embedded vascular structures in the brain also appear to have significant strain rates and cavitation. We simulated small embedded vascular structures with diameters as small as 0.6 mm. All of these embedded structures demonstrated significant cavitation and extremely high strain rates. Such strain rate focusing around vascular structures could be linked to the perivascular scarring unique to blast TBI (19). Another key clinical feature reported in blast TBI is the diffuse and rapid cerebral edema following severe blast exposure (14, 15). Cerebral edema can occur *via* many secondary / inflammatory mechanisms, however the early onset found in blast injury may reflect immediate (as opposed to secondary) injury to the smallest intracranial vessels—such as at the capillaries and blood brain barrier disruption *via* injury at the tight junctions of the endothelium, astrocytic foot processes, or basement membrane. Animal models have also demonstrated blood brain barrier disruption following blast exposure (24, 39). Our simulation does not model capillary dynamics (our smallest vessel is 0.6 mm—~60 times larger than capillaries). However, if the trend of strain focusing around and within small vascular structures continues to the microscale, then this same mechanism could hypothetically disrupt the blood brain barrier with resultant cerebral edema.

The periventricular scarring that is prominent in Perl et al.'s work (19) did not show as clear of an association with our measurements of cavitation and focal tissue strain-rates—specifically intraventricular cavitation was not prominent in our simulations until very large overpressures (see Figure 6). However, when intraventricular cavitation was detected it was associated with high adjacent periventricular tissue strain rates (not shown.) The lack of prominent intraventricular cavitation could be secondary to our ventricle design which were relatively open structures (that continued the entire length of the test object) as opposed to the relative confinement found in the ventricles of younger subjects—like that of Perl et al.'s subjects. Perhaps if our ventricles were reduced in volume to better reflect human anatomy, then more cavitation events may be captured (similar to the confinement effect within our vascular models). Such an investigation should be pursued in order to provide further insight for the hypothesis that cavitation and high periventricular strain rates could be linked to periventricular scarring.

Gray-white scarring also remains a prominent feature from Perl et al. (19). Our simulations do demonstrate some strain focusing at or near the gray-white interface (see Figure 4B)– as would be expected from traveling shear waves interacting with an abrupt interface of different shear properties. However, this pattern does not convincingly outline a single region of the gray-white interface.

### Interpretation of Mechanical Models

In our simulations, cavitation events predominately occur within the CSF spaces (near the regions of high tissue strain rates). Since the 1960's cavitation has been predicted to occur in brain injury (40) and this mechanism is also hypothesized to occur in blast injury, and is supported by predictions from advanced computer simulations of human heads blast injury (8, 10, 12, 41, 42) as well as experiments in human head surrogates (10, 11). Cavitation is a rapid expansion of a volume of tissue or fluid under high negative pressures *via* the formation of vapor bubbles or the nucleation of dissolved gasses. The process of cavitation has an inception phase (whereby bubbles form or grow), an expansion phase (where the bubble expands), and a collapsing phase (where vapor condensates or gas bubbles fragment). If collapsing takes place near a tissue border, a microjetting phase due to asymmetric collapsing also takes place. Bubble expansion (by directly displacing adjacent tissue) can lead to significant tissue strains and strain rates (43). The subsequent collapse of a bubble with formation of a microjet also leads to significant localized pressure and high strains and strain rates (43). It is important to note that cavitation can occur in aqueous tissue under high enough tensile forces, but experiments suggest that tissue would require more negative pressures than the relatively unconstrained intracranial fluids (37)—for example, CSF or blood.

There are multiple physical mechanisms by which cavitation can occur. One possibility is spallation—which can arise when pressure waves are transmitted across interfaces with different acoustic impedances (a function of density and speed of sound). Certainly, this is possible near the skull-CSF interface, but given that other intracranial tissue interfaces have a similar density and speed of sound, it would require very large pressure waves to induce spallation at tissue interfaces. However, there is another mechanism by which cavitation could be induced—resistance to accelerate a fluid adjacent to a moving interface. Such accelerations (or strain rates) are magnified between the interfaces of closely opposed interfaces—intravascular fluid, intrasulcal CSF, or CSF between skull and brain. Our simulations demonstrate cavitation events in both CSF and blood in regions of confinement and our vascular study implied that cavitation increased as the fluid confinement increased, i.e., sulci and vasculature (Figure 5E).

Importantly however, it is unclear in our simulation whether cavitation events are caused by the interface dynamics *via* inertial cavitation, or if cavitation events caused the increased strain rates localized near the cavitation events. Additionally, one must consider the specific cavitation model employed in the simulation to interpret our results. Our thresholds of cavitation were defined at a gauge pressure of −200 kPa for CSF and −300 kPa for gray and white matter. This threshold value for CSF is consistent with experimentally derived values −100 kPa absolute (−200 kPa gauge) and are commonly used in simulations (10, 44, 45). The threshold of −300 kPa within brain is consistent with experiments using gelatins as brain surrogates which show ~50% larger cavitation threshold as compared to an unconstrained fluid (37). In our simulations, below these cavitation inceptions pressures, elements stop supporting any additional tension while continuing to strain. Functionally, this response was similar to a yield point of a solid material where further deformation occurs without any increase in material tension. However, the dynamics of physical cavitation (as opposed to our model) are more complex. When a cavitation bubble forms, the internal pressure increases to approximately the vapor pressure of the surrounding fluid—functionally increasing the pressure within the bubble. This loss of tension would likely increase the dilatation rate (and diameter) of the cavitation bubble much beyond that predicted by our model. Additionally, in our simulation, the collapse of a cavitated region would produce neither a spike in local pressure or any microjetting phenomenon. In experimental studies, both bubble expansion and bubble collapse cause focal bursts of high pressure (10, 37, 43) which also deform adjacent soft tissue (43), and it is reasonable to assume these deformations (and perhaps high pressures) are injurious to the affected tissue. Because our relatively simple cavitation model does not resolve post inception bubble dynamics, we believe our simulation results are most valid at predicting the onset of cavitation. However, when predicting the full mechanical consequences of cavitation—either locally or on adjacent structures—we believe our predictions of the interface strains and strain rates adjacent to cavitation are conservative. Despite this conservative modeling of cavitation, it is important to highlight that there continues to be local maximums of strains rates at the mechanical interfaces within our human brain model.

### Limitations

There are several limitations to our computational approach. Firstly, our simulated head and skull—although scaled to capture human anatomic features—lacks many of the more complex features of intracranial anatomy (petrous and sphenoid ridges, curved calvarium, sub-cortical structures, etc). This simplicity limits interpretation to understand the mechanics of injury to key neuroanatomic structures and thus linking any symptoms / deficits to patterns found. Additionally, it is possible the choice of the anatomic slice which we essentially extruded to capture the “height” of a real human brain could limit the generalizability of our findings. However, firstly, the chosen slice did contain all of the anatomic structures of interest, and furthermore we believe this disadvantage did not outweigh the benefits of studying interface dynamics in an idealized geometry that would maximize physical insight into the problem. But further investigations with more complex models of anatomy will be necessary to further support our findings, as well as investigate if certain anatomic features / head anatomies have differential susceptibilities to blast exposures.

Another limitation is that our computational results are physical quantities such as pressure, strain, and strain rate, not biologic outcomes. Currently it is unclear if transient high pressures themselves alter cell behavior or can cause neuronal dysfunction, however there is older experimental data from blast lung that imply the high frequency components of pressure waves found in blasts (>3,000 Hz) may preferentially cause tissue injury over pressure waves with larger overpressures but slower frequencies (46). Additionally, blast overpressures onto cell cultures can reduce neuron viability (47), alter membrane permeability, increase synaptic protein loss (48), and lead to changes in long term potentiation (49). All these changes could result in clinical deficits, but direct evidence of these changes to clinical injury is lacking. Unlike from pressure, there exists more direct evidence that shear deformation injures brain tissue and is more clearly linked to traumatic brain injury and diffuse axonal injury (50). However, defining the actual “dose” of shear injury is more challenging because *in vitro* studies have shown that neurons are more sensitive to the direction of shear (relative to axonal direction) and amount of shear strain, while injury may not dependent on shear rate (51). Meanwhile, astrocyte viability after injury is likely secondary to strain rate and less influenced by shear amplitude or direction (52). What remains to be tested to further validate our hypothesis is if astroglial scarring patterns can be reproduced by high tissue strains or cavitation adjacent to cortical surfaces, and more importantly, if these scarring patterns are causative of clinical symptoms.

The results of any computational simulation of brain injury are dependent upon the material properties used for the various constituents. There exists very little data on the behavior of brain tissue under the high strain rates expected in blast injury. Our material models were based upon review of both human *in vivo* shear measurements as well as *ex vivo* animal models (20, 27, 35). In order to ensure results were not material-parameter-sensitive, we tested a range of shear properties by almost an order of magnitude—without significant changes in cavitation, strain or strain rate distributions. However, the results did have some dependence upon the skull dynamics. Increasing the stiffness of the skull reduce the overall strain in the brain material while increasing the strain rates nearest the skull. This is consistent with other simulations where skull flexure was shown to dramatically alter the intracranial stresses (5) and is supported by animal studies (6). However, the presence sulcal cavitation with adjacent peaks of brain strain rates was preserved in both sets of simulation. These simulations do highlight the importance of skull flexure modes in modifying the stains and strain rates within human TBI.

There are three specific limitations to our material modeling; we did not model the known rate-dependent shear response of brain tissue (53), brain tissue anisotropy (54), or any failure mechanisms of brain or skull (beyond cavitation). However, there currently is no consensus on the appropriate methodology to model these behaviors, nor has brain material been well-characterized within the high strain rate regimen expected in blast TBI, thus our focus was to use simplified, yet robust, computational models to understand the interplay of mechanical interfaces and clinical brain injury. But, given the robustness of findings across the large range of shear parameters tested, we anticipate the inclusion of these features would not negate our overall conclusions.

Lastly, traumatic brain injury (be it blast or blunt) is an incredibly complex and heterogenous injury. Several features, which dominate the management of these patients and pathophysiology, have been excluded from this model, specifically cerebral contusion, depressed skull fractures, intraventricular hemorrhage, penetrating injuries, etc. These pathologic features are resultant of large tissue strains (for example skull flexure of large magnitude such that the inner table of the skull impacts and possibly penetrates the brain.) Capturing such injury patterns accurately will require improved constitutive models and perhaps more importantly—mechanical failure models. Modeling materials after mechanical failure is challenging even for very well-characterized materials (steel, aluminum, etc.) while there do not exist any reliably developed and validated failure response models for any of the tissues within the human head. As such, our model (and all other computational models) are greatly limited in what severity of head injury they can completely model. However, assuming appropriate failure thresholds (such as our cavitation threshold) computational results could be a reasonable approximation of where cavitation could occur but also where we cannot fully resolve the resultant mechanics.

## Conclusions and Future Efforts

This computational experiment supports the hypothesis that blast TBI is an injury at the mechanical interfaces within the human head. With regard to supporting or refuting the existing mechanical hypotheses for mechanisms of primary blast injury, our model included direct transmission of pressure waves while also supporting that skull oscillations could alter intracranial stress. Our model addressed neither orifice transmission of pressure waves, nor did it attempt to model any thoracic transmission of pressure waves intracranially. Understanding this injury further will require accounting for the complex mechanics at these interfaces. Additionally, multiple future steps need to be taken in order to advance this knowledge to improve our ability to protect service members from blast TBI. Firstly, the phenomenology of these computer experiments must be validated. Because our computational test object can also be fabricated into a physical phantom which could be blast tested—we aim to experimentally capture these same phenomena under blast and blunt testing and the use of high-speed videography and particle- or pattern-tracking techniques. Secondly, more advanced models of brain tissue—to include anisotropy, strain rate dependence, and failure models—need to be developed and incorporated into large computational platforms such as the CTH-Sierra suite used in these simulations. Such a framework is scalable to simulate whole human head anatomy—with resolution of sub-millimeter vasculature, CSF spaces, and other anatomic features. Once this is accomplished, computational modeling could be used to define injury thresholds and optimize protective equipment to reduce injury from blast exposure. Lastly—there remains significant experimental efforts to correlate these mechanical strains / strain rates to biologic dysfunction in the multiple tissues effected to better understand pathophysiology which would be the path to improving care and developing protective pharmacologic agents.

## Data Availability Statement

The raw data supporting the conclusions of this article will be made available by the authors, without undue reservation.

## Ethics Statement

The imaging used to generate the idealized human head shape was built on by the author's (PI) medical imaging, therefore, full permission was received to publish this image.

## Author Contributions

AW, SM, and RM-A contributed to research design. CC, PE, JK, and SM contributed *via* performing computational simulations and finite element design. AW and SM analyzed results and contributed substantially to writing of document. All authors contributed to the article and approved the submitted version.

## Conflict of Interest

The authors declare that the research was conducted in the absence of any commercial or financial relationships that could be construed as a potential conflict of interest.
